# High engagement in nonpharmaceutical interventions and their associations with reduced COVID-19 among US college students

**DOI:** 10.1186/s12889-023-15916-0

**Published:** 2023-05-26

**Authors:** Marie-Claude Couture, Lindsey Walicek, Kelly L. L’Engle, Annette K. Regan

**Affiliations:** 1 School of Nursing and Health Professions, University of San Francisco, 2130 Fulton St, San Francisco, CA 94117 USA; 2 Fielding School of Public Health, University of California Los Angeles, Los Angeles, CA USA

**Keywords:** COVID-19, College students, Masks, Physical distancing, Young adults

## Abstract

**Background:**

Nonpharmaceutical interventions, including face mask-wearing, physical distancing, and avoidance of crowds and poorly ventilated spaces, have been widely recommended to limit the spread of SARS-CoV-2. To date, there is little data available on engagement in nonpharmaceutical interventions and COVID-19 in college students. Using a large sample of college students, we estimate the prevalence of engagement in mask-wearing, physical distancing, and avoidance of crowds/poorly ventilated spaces and their associations with COVID-19.

**Methods:**

A cross-sectional study was conducted (February–March 2021) using a college-wide online survey among students (*n* = 2,132) in California. Multiple modified poisson regression models assessed associations between mask-wearing indoors, physical distancing (both indoors or public settings/outdoors), avoidance of crowds/poorly ventilated spaces and COVID-19, controlling for potential confounders.

**Results:**

Fourteen percent (14.4%) reported a previous COVID-19 illness. Most students reported wearing masks consistently indoors (58%), and 78% avoided crowds/poorly ventilated spaces. About half (50%) reported consistent physical distancing in public settings/outdoor and 45% indoors. Wearing a mask indoors was associated with 26% lower risk of COVID-19 disease (RR = 0.74; 95% CI: 0.60,0.92). Physical distancing indoors and in public settings/outdoors was associated with a 30% (RR = 0.70; 95% CI: 0.56,0.88) and 28% (RR = 0.72; 95% CI: 0.58,0.90) decrease risk of COVID-19, respectively. No association was observed with avoidance of crowds/poorly ventilated spaces. The risk of COVID-19 declined as the number of preventive behaviors a student engaged in increased. Compared to those who did not engage in any preventive behaviors (consistently), students who consistently engaged in one behavior had a 25% lower risk (RR = 0.75; 95% CI: 0.53,1.06), those who engaged in two behaviors had 26% lower risk (RR = 0.74; 95% CI: 0.53,1.03), those who engaged in three behaviors had 51% lower risk (RR = 0.49; 95% CI: 0.33,0.74), and those who consistently engaged in all four behaviors had 45% lower risk of COVID-19 (RR = 0.55; 95% CI: 0.40,0.78).

**Conclusions:**

Wearing face masks and physical distancing were both associated with a lower risk of COVID-19. Students who engaged in more nonpharmaceutical interventions were less likely to report COVID-19. Our findings support guidelines promoting mask-wearing and physical distancing to limit the spread of COVID-19 on campuses and the surrounding communities.

**Supplementary Information:**

The online version contains supplementary material available at 10.1186/s12889-023-15916-0.

## Background

With the emergence of new SARS-CoV-2 variants and subvariants, coronavirus disease 2019 (COVID-19) continues to be a significant public health problem in the U.S. [1–3]. COVID-19 incidence has been highest in young adults [4], with more than 19 million cases so far among 18–29 years old in the US [5]. The prevalence of severe COVID-19 outcomes among young adults was high earlier in the pandemic [6, 7]. However, more recent estimates show that young adults aged 18 to 29 years old have a lower risk of hospitalizations and death [8]. Although most young adults recover from SARS-CoV-2 infection, almost one in five experienced post-COVID conditions (Long COVID) [9].

Institutions of higher education can serve as potential hot spots for transmission, where multiple COVID-19 clusters have been observed since the beginning of the pandemic [10–13]. Between the beginning of the pandemic and the end of May 2021, colleges and universities reported over 700,000 cases of COVID-19 across the country [14]. Despite this, immunization rates among young adults are suboptimal, especially among 18–24-year-olds [15], and current vaccines do not prevent SARS-CoV-2 transmission. As of April 2023, 66.8% of Americans aged 18 to 24 were considered fully vaccinated (two doses) ​​and 7.3% received the updated bivalent booster dose, the lowest percentages among adults [5]. In the face of suboptimal immunization rates, waning protection of the vaccine [16], and vaccines ineffective in blocking transmission, nonpharmaceutical COVID-19 preventive behaviors serve as important complementary tools in reducing infections in colleges and universities and transmission in surrounding communities.

Mask-wearing and physical distancing, and avoiding poorly ventilated spaces and crowds are nonpharmaceutical public health interventions recommended by the Centers for Disease Control and Prevention (CDC) and other public health organizations to reduce the risk of COVID-19 [17]. In a recent longitudinal study among a nationally representative U.S. sample, Andrasfay et al. [18] found that adults who were less likely to follow social-distancing guidelines (including avoiding large gatherings, going to bar, club, etc.) had a higher risk of COVID-19 diagnosis. Another large serial cross-sectional study conducted in the U.S. reported that a high proportion of mask-wearing combined with physical distancing was associated with a higher probability of SARS-CoV-2 transmission control [19]. A systematic review and meta-analysis of the evidence on the effectiveness of public health measures showed that mask-wearing and physical distancing reduced COVID-19 incidence by 53% and 25%, respectively [20]. A recent cluster-randomized trial of a community-level mask promotion intervention conducted in Bangladesh also demonstrated a 9.5% reduction in symptomatic COVID-19 prevalence [21]. This accumulation of scientific evidence supports that nonpharmaceutical interventions, especially mask-wearing and physical distancing, are useful preventive methods to reduce the risk of SARS-CoV-2 infections.

Despite these supportive data and the need for effective nonpharmaceutical interventions, few studies have examined engagement in preventive behaviors and COVID-19 in young adults or college students. One study among students living in dormitories at a large university in Wisconsin found that wearing masks during social events was associated with a lower risk of SARS-CoV-2 infections, but no statistically significant association was observed with engaging in social distancing [22]. Testing programs implemented in a Texas university found that most SARS-CoV-2 exposures involved one or both students not wearing masks [23]. Even if the scientific literature among college students is sparse, studies so far support previous findings showing that engagement in preventive behaviors can help reduce COVID-19 on campuses.

Given higher COVID-19 incidence and lower vaccination rates in young adults, understanding engagement in nonpharmaceutical interventions and their effects on COVID-19 in college students is essential to reduce transmission and protect the surrounding communities. Using a large sample of college students, we aimed to estimate the prevalence of engagement in mask-wearing, physical distancing, and avoidance of crowds/poorly ventilated spaces and their associations with COVID-19.

## Methods

### Study design, participants, and study population

A cross-sectional study was conducted (late February-early March 2021) at a university in an urban location considered one of the most diverse in the U.S. A university-wide sample was recruited by sending emails to the entire student community (*n* = 9,425). Reminder emails were sent after one, two, and three weeks to increase participation. A total of 2,532 students participated in the online survey (29.2%). Eligibility criteria included being aged 18 years or older, being currently enrolled as a student, and having the ability to give informed consent. All participants who consented to participate in the study were offered the possibility to enter a raffle to win participation prizes. The study was approved by the IRB of the Committee on Human Research at the university.

### Data collection and measures

Data were collected using a structured, online, self-administered questionnaire created using Qualtrics™. The questionnaire collected information on socio-demographics, COVID-19, preventive practices (i.e., mask-wearing and social distancing, avoidance of crowds/poorly ventilated spaces), and health risks. The questionnaire was pre-tested in a small sample of college students to assess the understanding, readability, wording, and clarity of the questions.

Preventive behavior exposure variables included frequency of engagement in face mask-wearing (indoors, with people not from your household), physical distancing (indoors with people not from your household and in public settings, including outside), and avoidance of crowds/poorly ventilated indoor spaces during the last month. Response categories for physical distancing, mask-wearing, and avoidance of crowds/poorly ventilated indoor spaces used a 5-point Likert Scale (all the time, most of the time, some of the time, a little of the time, not at all). These four variables were re-coded as “Consistently” (all the time) and “Inconsistently” (most of the time, some of the time, a little of the time, or not at all). SARS-CoV-2 is highly transmissible, and engaging in preventive behaviors consistently (i.e., all the time), such as mask-wearing, is recommended to protect against infection [24]. A new variable representing the number of preventive behaviors the students engaged consistently in was created by counting how many of these preventive behaviors each respondent reported practicing “Consistently” (all the time): 1, 2, 3, or all 4.

The primary outcome, ever being diagnosed with COVID-19 illness, was assessed using the following response categories: “yes, I was diagnosed by a healthcare provider or tested positive'', “no, I was not diagnosed but I believe I had COVID-19”, “no, I have never had COVID-19”. This variable was recoded as “yes” (yes, I was diagnosed/ no, but I believe I had COVID-19) and “no” (no, I never had COVID-19). Since testing was limited, especially early in the pandemic, those who responded that they were not officially diagnosed but believed they had COVID-19 were considered to have had COVID-19 illness. In our analyses, those who believed they had COVID-19 but were not formally diagnosed had similar characteristics to those officially diagnosed with COVID-19. Moreover, according to a seroprevalence study conducted at a similar time (April 2021 to June 2021) in California, the seroprevalence from SARS-Cov-2 infection alone was 18% (95% CI, 14%–22%) in adults and 26% (95% CI, 19%–32%) in children [25]. In our study, only 6.8% (*n* = 140) of the participants had received a COVID-19 diagnosis. However, 14.4% had either received a diagnosis or believed they had COVID-19, which is closer to the seroprevalence estimate in California at that time.

Other covariates or potential confounders measured included sociodemographics and health risk variables. Sociodemographics included age (continuous), gender (male, female, trans/queer/gender, not included), race/ethnicity (White, Asian, Black, Latinx/Hispanic, other, multiple race/ethnicity), marital status (never married, married/live with a partner, separated/divorced/widowed), sexual orientation (straight, gay, lesbian, bisexual, queer, other), religion (Christian-Catholic, Christian-non Catholic, Muslim, Buddhism, Hinduism, Judaism, other religion, unaffiliated), political ideology (conservative/very conservative, center, liberal, very liberal), number of people living in the household (continuous), and being an essential worker (yes, no). Income level was assessed by asking participants how they were feeling about their income: “living comfortably,” “getting by on income,” “finding it difficult”, or “finding it very difficult”. Students were asked if they have a health condition that makes them more susceptible to severe illness from COVID-19 according to the CDC (yes, no, unsure). Health conditions included asthma, Type 1 diabetes, Type 2 diabetes, chronic heart disease, hypertension or high blood pressure, cancer, chronic kidney disease, liver disease, COPD, Down syndrome, cystic fibrosis, cerebrovascular disease, and Sickle cell disease. Participants were also asked if they live with someone who has been diagnosed with one of these health conditions (yes, no).

### Statistical analysis

Statistical analyses were conducted using STATA/IC 16.1 (STATA, College Station, TX). Recommendations regarding nonpharmaceutical interventions varied by state, and therefore analyses were restricted to only respondents living in California (*n* = 2,132). Chi-square and Fisher's Exact tests were conducted to examine the associations between previous COVID-19 disease and engagement in preventive behaviors (mask-wearing, physical distancing, and avoidance of crowds/poorly ventilated spaces), and also sociodemographics and health risks. Multiple modified poisson regression models assessed associations between the exposure variables i) mask-wearing indoors, ii) physical distancing (indoors and public settings/outdoors), and iii) avoidance of crowds/poorly ventilated spaces and previous COVID-19 disease. Confounding variables were selected for inclusion in adjusted models based on identification as potential confounders using both theoretical and empirical methods [26, 27]. We used a theoretical strategy based on previously published scientific literature and underlying causal structure to identify potential confounders, excluding instrumental variables and mediators. Then, we tested if the potential confounding variables were associated with the independent variable (primary exposure) and also independently associated with the outcome using bivariate analyses. We also tested if the coefficient for the effect of the independent variable (or primary exposure) on the outcome changed significantly when the potential confounding variables were added to the multiple regression models. Akaike information criterion (AIC) and Bayesian information criterion (BIC) were used to assess the adequacy of the multiple modified poisson models. Based on this strategy, the final models were adjusted for race/ethnicity, age, gender, and living with someone who has a high-risk health condition.

## Results

A total of 2,132 participants living in California responded to the survey with a mean age of 24.8 years old (SD 7.12 years). The majority of participants were female (74%), 23% were male, and 3% were trans/queer or reported that their gender was not included (Table 1). One-third of the students reported being Asian (33%), 28% were White, 16% were multi-race, 15% were Latinx/Hispanic, 5% were Black, and 4% chose other race/ethnicity. Most students were straight (77%), and 22.6% were LGBQ or reported other sexual orientations. Most students were Christian (40.7%) or religiously unaffiliated (42.5%). More than half of the students were politically liberal or very liberal (72.5%). Students reported living in a household with a mean of 3.5 people. Close to a third (30.4%) of the participants reported being essential workers and 30.8% reported finding it difficult or very difficult to live on their income. Sixteen percent (15.8%) reported having a health condition putting them at high risk of having severe COVID-19 and 29.5% lived with someone with a high-risk condition.

**Table 1:** Prevalence of sociodemographic, health risk factors, and preventive behaviors, and bivariate associations with previous COVID-19 illness among college students (*n* = 2,132)

Characteristics	Prevalence of characteristics	Prevalence of COVID-19 disease	p-value*
	n (%)	n (%)	
Total	2,060	297 (14.4)	
**Sociodemographic**			
Age: mean = 24.8			0.891
*18–19 years old*	326 (15.5)	42 (13.4)	
*20–29 years old*	1,426 (67.7)	204 (14.8)	
*30–39 years old*	242 (11.5)	34 (14.6)	
*40* +	114 (5.4)	14 (12.8)	
Gender			0.989
*Male*	497 (23.30)	69 (14.5)	
*Female*	1,575 (73.9)	220 (14.4)	
*Trans/Queer/Not included*	60 (2.8)	8 (13.8)	
Race/ethnicity			** < 0.001**
*White*	594 (27.9)	83 (14.5)	
*Asian*	697 (32.8)	57 (8.4)	
*Black*	97 (4.6)	11 (11.7)	
*Latinx/Hispanic*	328 (15.4)	70 (21.9)	
*Other*	81 (3.8)	7 (22.8)	
*Multiple race/ethnicity*	331 (15.6)	58 (18.4)	
Marital status			0.639
*Never married*	1,562 (79.2)	212 (14.0)	
*Married/live with partner*	365 (18.5)	47 (13.3)	
*Separated/divorced/widowed*	45 (2.28)	8 (18.6)	
Sexual orientation			0.876
*Straight*	1,521 (77.4)	212 (14.3)	
*Gay*	70 (3.6)	7 (10.5)	
*Lesbian*	36 (1.8)	4 (11.8)	
*Bisexual*	206 (10.5)	26 (13.0)	
*Queer*	87 (4.4)	10 (12.4)	
*Other*	45 (2.3)	8 (18.2)	
Religion			0.210
*Christian-Catholic*	573 (27.1)	87 (15.5)	
*Christian-Non Catholic*	268 (13.6)	42 (16.1)	
*Muslim*	50 (2.5)	10 (20.4)	
*Buddhism*	88 (4.5)	9 (10.2)	
*Hinduism*	56 (2.9)	8 (14.8)	
*Judaism*	42 (2.1)	9 (21.4)	
*Other*	54 (2.7)	7 (14.0)	
*Unaffiliated*	837 (42.5)	95 (11.9)	
Political ideology			0.701
*Conservative/very conservative*	103 (4.9)	18 (18.6)	
*Center*	481 (22.7)	66 (14.5)	
*Liberal*	1,050 (49.5)	145 (14.2)	
*Very liberal*	488 (23.0)	67 (14.2)	
Number of people in household: mean = 3.5			0.120
Essential worker			0.494
*Yes*	649 (30.4)	96 (15.2)	
*No*	1,484 (69.6)	201 (14.1)	
Feeling about income			0.528
*Comfortable*	569 (29.0)	70 (12.6)	
*Getting by*	792 (40.3)	108 (14.1)	
*Difficult*	406 (20.7)	58 (14.7)	
*Very difficult*	198 (10.1)	32 (16.7)	
**Health Risks**			
Has high-risk health condition			0.998
*Yes*	305 (15.8)	44 (14.4)	
*No*	1,753 (81.2)	253 (14.4)	
Live with someone with high-risk health condition			**0.002**
*Yes*	629 (29.5)	111 (18.2)	
*No*	1,504 (70.5)	186 (12.8)	
**Preventive Behavior exposures**			
Wear face mask indoors			**0.017**
*Consistently*	1,190 (58.0)	144 (12.5)	
*Inconsistently*	862 (42.0)	136 (16.3)	
Physical distance indoors			
*Consistently*	922 (44.9)	108 (12.0)	**0.015**
*Inconsistently*	1,132 (55.1)	172 (15.8)	
Physical distance outdoors/in public settings			**0.009**
*Consistently*	1,025 (50.0)	121 (12.1)	
*Inconsistently*	1,028 (50.0)	159 (16.1)	
Avoid crowds/poorly ventilated spaces			
*Consistently*	1,598 (77.9)	207 (13.3)	0.067
*Inconsistently*	454 (22.1)	73 (16.8)	

^*^p-values comparing the prevalence of COVID-19 illness across participants’ characteristics

Bolded: *p* < 0.05

Fourteen percent (14.4%) reported previous COVID-19 diagnosis or believed they had COVID-19 (*n* = 297) (Table 1). The majority of students reported wearing face masks indoors and avoiding crowds/poorly ventilated spaces all the time (58% and 78%, respectively). Forty-five percent of students reported engaging in physical distancing indoors consistently and 50% physical distancing outdoors/in public settings.

COVID-19 was lower among students who reported wearing face masks indoors consistently (12.5%) compared to those who used face masks inconsistently (16.3%) (*p* = 0.017) (Table 1). Students who engaged in physical distancing indoors consistently had lower COVID-19 illness than those who did not (12.0% and 15.8% respectively; *p* = 0.015). COVID-19 was less common among respondents who did engage in physical distancing outdoors/in public settings consistently (12.1%) compared to those who did not (*p* = 0.009). The prevalence of COVID-19 was 13.3% among participants who avoided crowds and poorly ventilated indoor spaces consistently compared to 16.8% among those who did not (*p* = 0.067).

Prevalence of previous COVID-19 illness also differed by race/ethnicity (Table 1). Prevalence was 22.8% among students who reported “Other” race/ethnicity, 21.9% among Latinx/Hispanic students, 18.4% among multi-race students, 14.5% among White students, 11.7% among Black students, and only 8.4% among Asian students (*p* < 0.001). COVID-19 was highest among students who live with someone who has a health condition putting them at high risk of having severe COVID-19 (18.2%) compared to those who did not (12.8%; *p* = 0.002). No statistically significant differences were observed with other sociodemographic and health risk variables.

Table 2 shows the sociodemographic and health risk factors associated with consistent engagement in the different preventive behaviors in the bivariate associations. Older age was associated with consistent mask-wearing indoors (*p* < 0.001), physical distancing indoors (*p* < 0.001), and in public settings/outdoors (*p* < 0.001), but not avoidance of crowds and poorly ventilated spaces. Students who identified as trans, queer or reported that their gender was not included were more likely to avoid crowds and poorly ventilated spaces (*p* < 0.001) compared to male and female students. Prevalence of consistent engagement in mask-wearing indoors and physical distancing (indoor and public settings/outdoors) were higher among participants from other race/ethnicity, Black, and Latinx/Hispanic students (*p* < 0.001). Students who were not married were less likely to consistently engage in mask-wearing indoors and physical distancing (indoor and public settings/outdoors) (*p* < 0.001). Physical distancing indoors was higher among Muslim students (70.0%) and Hindu students (56.4%) (*p* = 0.006). Buddhist and Muslim students were more likely to report consistently avoiding crowds and poorly ventilated spaces (87.5% and 84%, respectively; *p* < 0.001) (Table 2). The prevalence of consistent engagement in all the preventive behaviors was higher among participants who reported being liberal or very liberal (*p* < 0.001). Essential workers were more likely to engage in physical distancing indoors (*p* = 0.032) and in public settings/outdoors (*p* = 0.004). Students who reported living with someone with a high-risk health condition were more likely to avoid crowds and poorly ventilated spaces (*p* = 0.001).

**Table 2:** Sociodemographic and health risk factors associated with consistent engagement in preventive behaviors in bivariate analyses (*n* = 2,132)

Characteristics	Wear face mask indoors	*p-value*	Physical distance indoors	*p-value*	Physical distance outdoors/in public settings	*p-value*	Avoid crowds/poorly ventilated spaces	*p-value*
	n (%)		n (%)		n (%)		n (%)	
Total	1,190 (58.0)		922 (44.9)		1,025 (50.0)		1598 (77.0)	
**Sociodemographic**								
Age: mean = 24.8		** < 0.001**		** < 0.001**		** < 0.001**		0.680
*18–19 years old*	174 (55.4)		139 (44.3)		151 (48.2)		252 (80.3)	
*20–29 years old*	759 (55.6)		572 (41.9)		652 (47.7)		1,052 (77.1)	
*30–39 years old*	162 (68.1)		137 (57.6)		143 (60.1)		188 (79,3)	
*40* +	61 (74.4)		47 (56.6)		49 (59.0)		62 (74.7)	
*50 years or older*	20 (71.4)		21 (75.0)		17 (60.7)		22 (78.6)	
Gender		0.231		0.221		0.446		** < 0.001**
*Male*	278 (58.2)		213 (44.6)		235 (49.2)		342 (71.7)	
*Female*	873 (57.5)		677 (44.5)		757 (49.8)		1,207 (79.5)	
*Trans/Queer/Not included*	38 (69.1)		31 (56.4)		32 (58.2)		49 (89.1)	
Race/ethnicity		** < 0.001**		** < 0.001**		** < 0.001**		** < 0.001**
*White*	333 (57.9)		251 (43.6)		249 (43.2)		375 (65.1)	
*Asian*	369 (55.0)		274 (40.8)		345 (51.4)		586 (87.3)	
*Black*	58 (63.0)		45 (48.9)		52 (56.5)		81 (89.0)	
*Latinx/Hispanic*	197 (61.6)		154 (48.1)		173 (54.1)		254 (79.4)	
*Other*	59 (79.7)		50 (67.6)		49 (66.2)		58 (78.4)	
*Multiple race/ethnicity*	169 (53.7)		144 (45.7)		153 (48.6)		240 (76.2)	
Marital status		**0.007**		** < 0.001**		** < 0.001**		0.806
*Never married*	876 (56.1)		668 (42,8)		751 (48.1)		1,216 (78.0)	
*Married/live with partner*	232 (64.1)		189 (51.9)		205 (56.3)		278 (76.6)	
*Separated/divorced/widowed*	31 (68.9)		29 (64.4)		25 (55.6)		34 (75.6)	
Sexual orientation		0.931		0.175		0.882		0.836
*Straight*	872 (56.1)		665 (43.8)		758 (50.0)		1,172 (77.3)	
*Gay*	40 (57.1)		31 (44.3)		32 (45.7)		54 (77.1)	
*Lesbian*	23 (63.9)		22 (61.1)		21 (58.3)		30 (83.3)	
*Bisexual*	119 (57.8)		95 (46.1)		100 (48.5)		160 (77.7)	
*Queer*	54 (62.1)		47 (54.0)		42 (48.3)		72 (82.8)	
*Other*	27 (60.0)		41 (46.7)		22 (48.9)		35 (77.8)	
Religion		0.776		**0.006**		0.165		** < 0.001**
*Christian-Catholic*	321 (56.2)		250 (43.7)		288 (50.4)		469 (82.1)	
*Christian-Non Catholic*	159 (59.3)		108 (40.3)		129 (48.1)		189 (70.8)	
*Muslim*	32 (64.0)		35 (70.0)		31 (62.0)		42 (84.0)	
*Buddhism*	54 (61.4)		40 (45.5)		49 (55.7)		77 (87.5)	
*Hinduism*	29 (52.7)		31 (56.4)		33 (60.0)		47 (45.5)	
*Judaism*	28 (66.7)		22 (52.4)		22 (54.2)		25 (59.5)	
*Other*	31 (58.5)		26 (49.1)		30 (56.6)		44 (83.0)	
*Unaffiliated*	483 (57.7)		369 (44.1)		394 (47.1)		632 (75.5)	
Political ideology		**0.008**		** < 0.001**		** < 0.001**		** < 0.001**
*Conservative/very conservative*	42 (43.3)		28 (28.6)		30 (30.6)		53 (54.6)	
*Center*	260 (56.4)		192 (41.7)		229 (49.7)		349 (75.7)	
*Liberal*	588 (58.1)		442 (43.6)		498 (49.2)		798 (78.8)	
*Very liberal*	292 (61.9)		251 (53.2)		260 (55.1)		389 (82.4)	
Number of people in household: mean = 3.5		0.496		0.440		0.185		**0.021**
Essential worker		0.296		**0.032**		**0.004**		0.995
*Yes*	354 (56.3)		260 (41.3)		284 (45.2)		489 (77.9)	
*No*	836 (58.8)		662 (46.5)		741 (52.0)		1,109 (77.9)	
Feeling about income		0.873		0.454		**0.008**		
*Comfortable*	324 (57.1)		420 (42.3)		262 (46.2)		429 (75.5)	0.389
*Getting by*	454 (75.4)		357 (45.1)		395 (49.9)		623 (78.7)	
*Difficult*	240 (59.3)		188 (46.4)		200 (49.4)		312 (77.4)	
*Very difficult*	117 (59.4)		94 (47.7)		119 (60.4)		159 (80.7)	
**Health Risks**								
Has high-risk health condition		0.249		0.149		0.601		
*Yes*	188 (61.0)		150 (48.7)		158 (51.3)		242 (78.6)	
*No*	1,002 (57.5)		772 (44.2)		866 (49.7)		1,355 (78.8)	
Live with someone with high-risk health condition		0.821		0.147		0.135		**0.001**
*Yes*	352 (58.4)		286 (47.4)		317 (52.5)		498 (82.4)	
*No*	838 (57.8)		636 (43.9)		798 (48.9)		1,100 (76.0)	

Bolded: *p* < 0.05

**Fig. 1:**
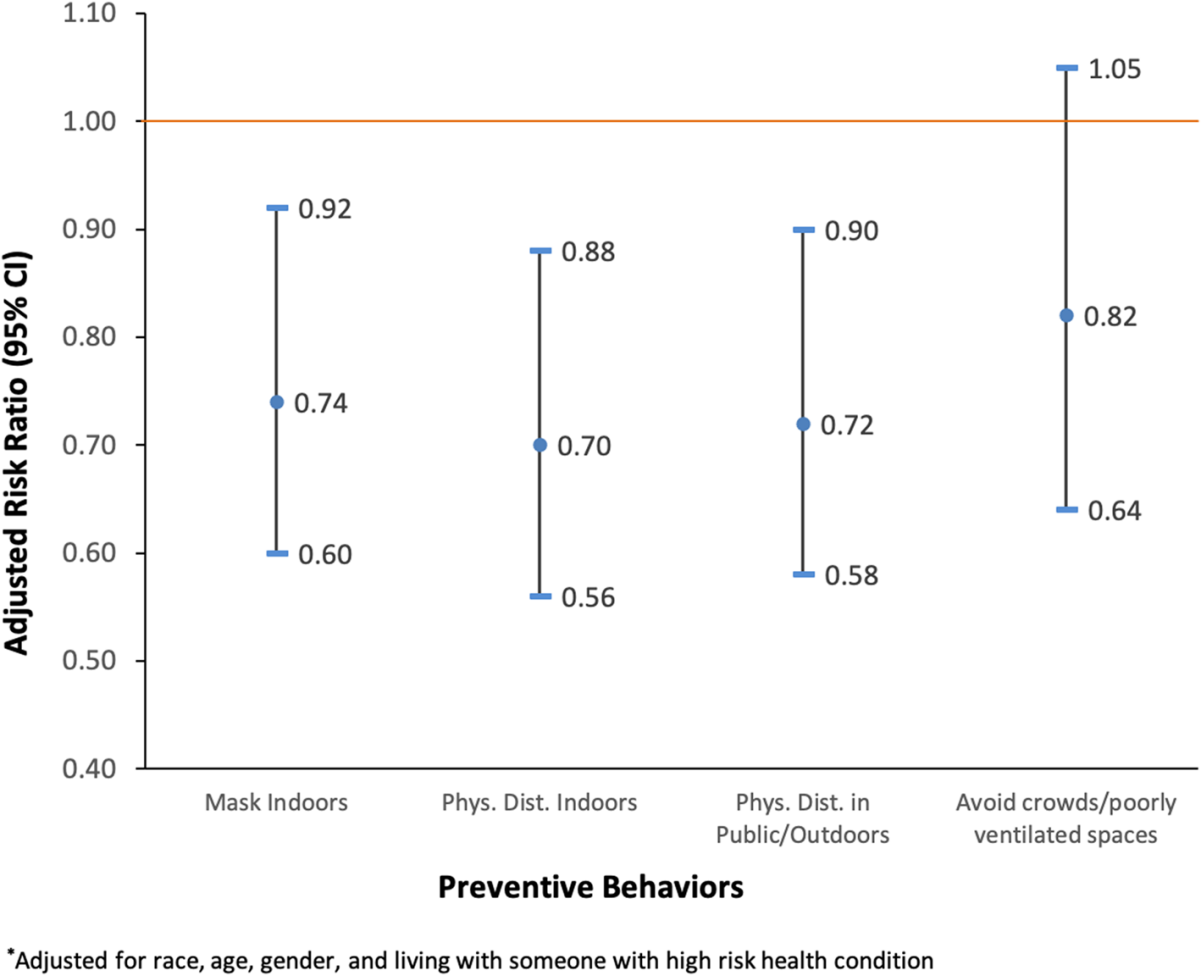
Adjusted risk ratios of COVID-19 illness by engagement in preventive behaviors among college students

**Fig. 2:**
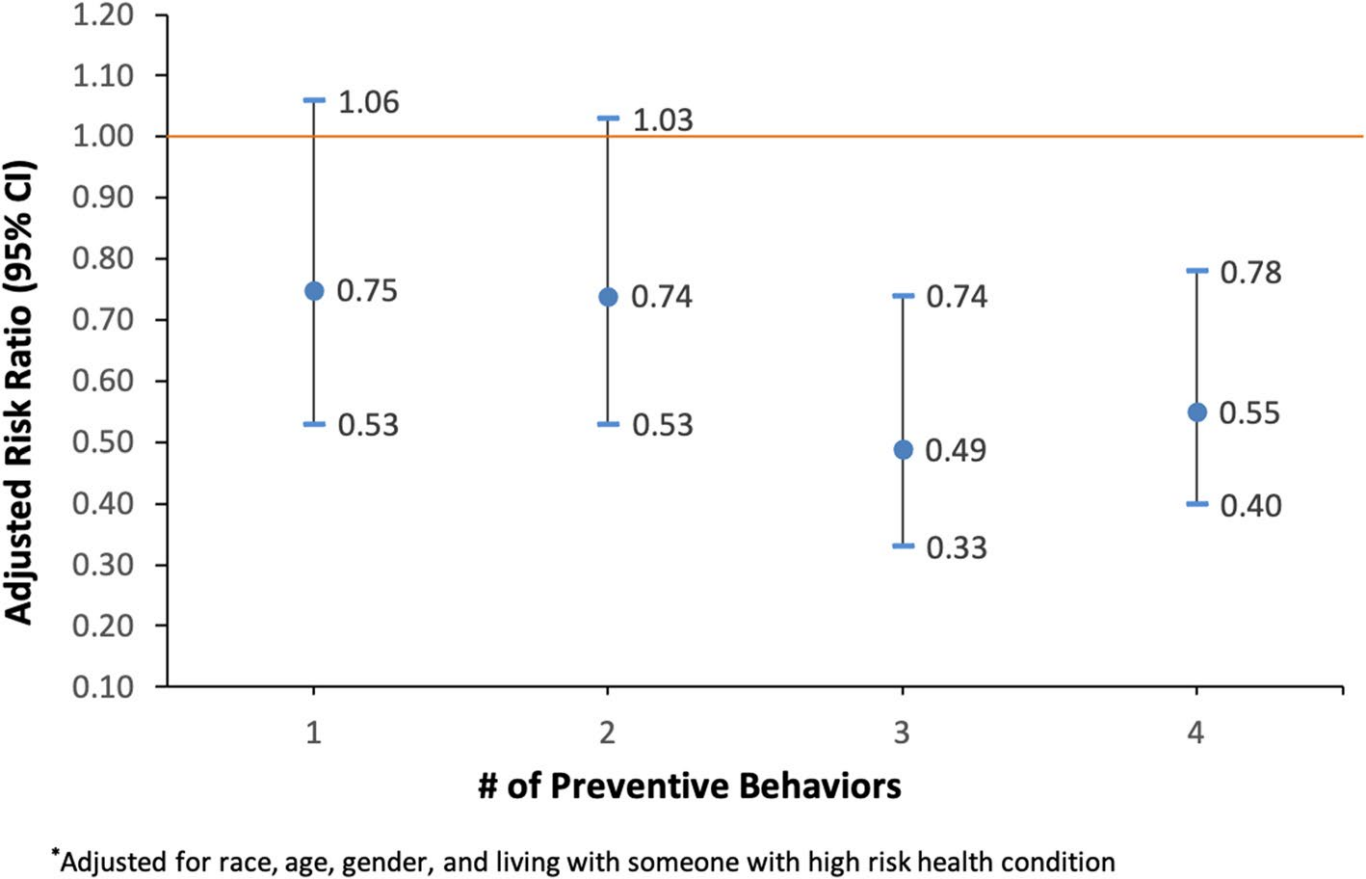
Adjusted risk ratios of COVID-19 illness by the number of preventive behaviors engaged in

In multiple regression models, consistent mask-wearing indoors was associated with a 26% lower risk of COVID-19 illness (RR = 0.74; 95% CI: 0.60,0.92; Fig. 1). Consistently engaging in physical distancing indoors and in public settings/outdoors was associated with a 30% (RR = 0.70; 95% CI: 0.56,0.88) and 28% (RR = 0.72; 95% CI: 0.58,0.90) decrease in the risk of COVID-19 illness, respectively (Fig. 1). ​​No statistically significant association was observed with avoidance of crowds and poorly ventilated spaces and COVID-19. COVID-19 declined as the number of preventive behaviors a student engaged in increased. Compared to those who did not engage in any preventive behaviors (consistently), students who consistently engaged in one behavior had a 25% lower risk of COVID-19 (RR = 0.75; 95% CI: 0.53,1.06), those who consistently engaged in two behaviors had 26% lower risk (RR = 0.74; 95% CI: 0.53,1.03), those who consistently engaged in three behaviors had 51% lower risk (RR = 0.49; 95% CI: 0.33,0.74), and those who consistently engaged in all four behaviors had 45% lower risk of COVID-19 (RR = 0.55; 95% CI: 0.40,0.78) (Fig. 2).

Lastly, we conducted sensitivity analyses where all the preventive behaviors exposure variables were recoded as “Consistently” combining both “all the time” and “most of the time” responses, and as “Inconsistently” combining “some of the time”, “a little of the time”, and “not at all” responses. In the multiple modified poisson regression models, results were similar but some associations were not statistically significant, most likely due to lower statistical power (Supplementary Table 1). The survey was conducted in the Spring of 2021, earlier in the epidemic when the CDC and other health organizations strongly recommended that people engage in these preventive behaviors to protect against COVID-19. Thus, the prevalence of participants engaging in these preventive behaviors “all the time” and “most of the time” was high and fewer respondents answered “some of the time”, “a little of the time” or “not at all”, leading to very small samples in these categories. Combined with a low prevalence of COVID-19 illness in some categories, the lack of statistical significance is likely the result of the lower statistical power to detect associations.

## Discussion

Our research showed a lower prevalence of COVID-19 disease among college students who consistently engaged in nonpharmaceutical preventive behaviors, including wearing masks indoors, physical distancing indoors, or physical distancing outdoors/in public settings. However, no association was observed between avoidance of crowds and poorly ventilated indoor spaces and COVID-19. Overall, the more preventive behaviors that college students engaged in, the less likely they were to report a COVID-19 illness. However, the risk of COVID-19 decreased only after a threshold was reached: students who consistently engaged in three or four preventive behaviors were 51% and 45% less likely respectively to report previous COVID infection compared to those who did not engage in any preventive behaviors. Even if the associations did not reach statistical significance, a similar trend in reduction was observed for engaging in only one or two preventive behaviors [28]. These findings may highlight the importance of engaging in several preventive behaviors in order to add layers of protection [29, 30].

The current literature on engagement in physical distancing and mask-wearing and COVID-19 largely focuses on the general adult population with limited research on young adults and college students. However, our findings are consistent with previous studies conducted in the general population supporting the effectiveness of mask-wearing to protect against COVID-19, particularly in indoor settings [19–21]. Lower SARS-CoV-2 transmission was also observed in states and counties with mask mandates [31, 32]. Our results on physical distancing support previous studies that have observed a decreased risk of COVID-19 with more frequent social distancing [18, 20]. Individuals who live in communities with more social distancing also are at lower risk of COVID-19 [33]. Few studies have examined a possible dose–response effect of engaging in multiple preventive behaviors on COVID-19, although Andrasfay et al. [18] found that each additional high-risk behavior individuals engaged in (e.g., not wearing a face mask, close contact with non-household members, attending large gatherings, going to bar, club, etc.) was associated with a 9% higher risk of COVID-19 diagnosis. Our findings further support mask-wearing and physical distancing guidelines, especially indoors and particularly in environments frequented by young adults such as college and university settings, to protect against COVID-19.

Our study was conducted in a very diverse population of college students, which is a strength of the study. However, our study has other limitations that should be considered when interpreting the results. The study used a cross-sectional study design, which does not allow the determination of temporality or a causal relationship between engagement in preventive behaviors and COVID-19 disease, implying a risk of potential reverse causality. Participants were sampled from one university using a nonprobabilistic sampling method that may lead to selection bias. Yet, the socio-demographic characteristics of our sample were representative of the entire university population where the study was conducted. The age of our participants was similar to the US college student population. Although, our sample included a slightly higher proportion of female students, which might be less representative of the general US college student population. This could mean that prevalence estimates of engagement in nonpharmaceutical preventive behaviors differ from the general population of college students. However, we would not expect the effect of nonpharmaceutical preventive behaviors to vary based on population characteristics since the biological mechanisms behind the association between engagement in preventive behaviors and protection against SARS-CoV-2 infection should be consistent across populations. The data collected were self-reported and may be subject to recall bias and social desirability bias. Nonetheless, this would lead to more conservative estimates, biasing our results towards the null. Due to the limited access to testing at the time of our study, our outcome measure of COVID-19 disease included both participants who were diagnosed and those who did not have an official diagnosis but believed they had COVID, which could have led to misclassification. The prevalence of COVID-19 disease in our study was similar to the seroprevalence estimate of SARS-CoV-2 due to infection in California at that time [25], supporting the inclusion of participants who believed they had COVID but without a medical diagnosis. However, the prevalence of COVID-19 reported in our study is likely underestimated as some participants could have been infected but asymptomatic. Residual confounding by other potential confounders unmeasured and uncontrolled in the analysis is another limitation. Furthermore, no information was collected about the types of face masks used. The questions on mask-wearing and physical distancing referred to broad environments such as "indoors" and "outdoors" and thus do not capture potential nuances in students' preventive behaviors; a finer assessment of preventive practices under certain situations (e.g., in dorms or homes, when eating with others, in classrooms or gyms) would permit more focused analyses that would provide information about unique situations that might contribute more or less to transmission.

Future research should include mixed-research approaches to better understand why, when, where, and with whom young adults engage in preventive behaviors to protect themselves against COVID-19. Understanding these motivations will help inform effective preventive interventions that support increasing adherence to engaging in mask-wearing and physical distancing among young adults. Longitudinal research studies are also needed to establish the temporality of the association between engagement in preventive behaviors and COVID-19 in college students and ascertain causality. Further studies should also include a larger more representative sample of U.S. college students to improve external validity.

Endorsing policies that encourage engagement in these nonpharmaceutical interventions on campus combined with vaccination could help reduce COVID-19 transmission among college students, and potentially to their families and friends, and the surrounding communities. College and university policies should promote wearing a face mask indoors and physically distancing while on campus when community transmission levels are higher as recommended by the CDC [34]. A campus-wide policy could be championed by both student health services and student ambassadors who, as trusted healthcare providers and as peers, would be effective in persuading college students to comply with the policy. State- and city-wide COVID-19 prevention policies encouraging engagement in these nonpharmaceutical interventions and vaccination aligned with campus-wide policies for college students and young adults would benefit students and their families outside of the campus environment. Social media and other communication platforms could be used to spread accurate information messages encouraging engagement in physical distancing and wearing a face mask indoors, as well as highlighting the importance of protecting the students’ family members, friends, and surrounding community.

## Conclusion

Our findings support promoting physical distancing and mask-wearing in indoor settings, in combination with COVID-19 vaccination, as additional measures to further protect college students against COVID-19 disease. Our research helps inform strategies for college and university campuses—especially those campuses in states with low vaccination rates and higher community transmission—in considering ways to move forward with curriculum planning. SARS-CoV-2 is expected to continue circulating and the US will likely see other surges. Scientists also believe the emergence of new variants is inevitable [35]. Thus, nonpharmaceutical interventions against SARS-CoV-2 infection combined with vaccination continue to be essential for the long-term management of the COVID-19 pandemic and should be included in plans to prepare for future epidemics.

## Supplementary Information

**Additional file 1.**

**Additional file 2.**

## Data Availability

The dataset is not publicly available due to IRB requirements but is available upon request to the corresponding authors.

## Abbreviations

CDC: Centers for Disease Control and Prevention
COPD: Chronic Obstructive Pulnomary Disease
COVID-19: Coronavirus disease 2019
IRB: Institutional Review Board
LGBQ: Lesbian, gay, bisexual, queer
