# Supplementary material for: High engagement in nonpharmaceutical interventions and their associations with reduced COVID-19 among US college students
Source: BMC Public Health. 2023 May 26;23:971. doi: 10.1186/s12889-023-15916-0 (PMC10214357; doi:10.1186/s12889-023-15916-0)
Supplement: Supplementary file 1 — Additional file 1. [file 12889_2023_15916_MOESM1_ESM.pdf]

Student Covid-19 Survey

Start of Block: Demographics

Q2 **Please tell us more about yourself:** Are you currently enrolled as a USF student?

- Yes, full-time (1)
- Yes, part-time (2)
- Not currently enrolled or on leave (3)
- Not included here, please specify: (4) ____

Q3 What school or college is your major or program located in?

- College of Arts and Sciences (1)
- School of Education (2)
- School of Law (3)
- School of Management (4)
- School of Nursing and Health Professions (5)

Q4 Are you an undergraduate or graduate student?

- ○ First year undergraduate (1)
- ○ Second year undergraduate (2)
- ○ Third year undergraduate (3)
- ○ Fourth year undergraduate (4)
- ○ Undergraduate student with five or more years (5)
- ○ Graduate student (master) (6)
- ○ Graduate student (doctoral) (7)
- ○ Not included here, please specify: (8) ____

Q5 How old are you?

Enter your age in years below

____

Q6 What is your race/ethnicity? (Check all that apply)

- ▢ Asian/Asian American (1)
- ▢ Pacific Islander/ Native Hawaiian (2)
- ▢ Black/ African American (3)
- ▢ Latina/o/x/Hispanic (4)
- ▢ Native American/ First Nation/ Alaska Native (5)
- ▢ Arab/ Middle Eastern (6)
- ▢ White/ Caucasian (7)
- ▢ Other, please specify (8) ____

Q7 What is your gender?

- ○ Male (1)
- ○ Female (2)
- ○ Trans male/female-to-male (FTM) (3)
- ○ Trans female/male-to-female (MTF) (4)
- ○ Nonbinary, gender nonconforming, or genderqueer (5)
- ○ Gender not included (9)

Q8 Which of the following best describes your current living situation?

- ◯ I live on campus in USF housing (1)
- ◯ I live off-campus by myself (2)
- ◯ I live off-campus with roommates (3)
- ◯ I live with my partner or spouse (4)
- ◯ I live with my parents or other family members (5)
- ◯ Not included here, please specify (6) ____________________

*

Q9 How many total people (adults and children) currently live in your household? (A household could be a dorm room, an apartment, a single family home, etc).

____

Q10 How many individuals in each age group live with you? Enter 0 if none in age group.

- ◯ 0 - 5 (1) ____________________
- ◯ 6 - 17 (2) ____________________
- ◯ 18 - 24 (3) ____________________
- ◯ 25 - 29 (4) ____________________
- ◯ 30 - 44 (5) ____________________
- ◯ 45 - 64 (6) ____________________
- ◯ 65 - 74 (7) ____________________
- ◯ 75 + (8) ____________________

Q11 In which country do you currently live?

- ○ United States (1)
- ○ Outside the US (Please specify both City, Country) (2) ____

*

Q12 What is the zip code of your current residence?

____

Q13 How do you describe your political ideology?

- ○ Very conservative (1)
- ○ Conservative (2)
- ○ Center (3)
- ○ Liberal (4)
- ○ Very liberal (5)

Q14 Do you have a health condition that may make you more susceptible to severe illness from COVID-19?

According to the CDC, these conditions include asthma, Type 1 diabetes, Type 2 diabetes, chronic heart disease, hypertension or high blood pressure, cancer, chronic kidney disease,

liver disease, COPD, Down syndrome, Cystic fibrosis, Cerebrovascular disease, Sickle cell disease.

- Yes (1)
- No (2)
- Unsure (3)

Q15 Has anyone else that you live with been diagnosed with one of these conditions?

- Yes (1)
- No (2)
- Unsure (3)

Q16 Are you currently employed?

- Yes, full-time (1)
- Yes, part-time (5)
- No (6)

Q17 Are you considered an essential worker, meaning you are still required to work outside your home during the COVID-19 pandemic?

- Yes (1)
- No (2)
- Unsure (3)

Q18 Which best describes your main workplace?

- ○ Hospital, health or dental clinic, doctor's office (1)
- ○ Health or social services department such as a local DPH or Housing (5)
- ○ Caregiver in a nursing home, care facility, or home health provider (7)
- ○ Teacher, educational staff, or childcare provider (9)
- ○ Services professional such as restaurant, market, store; delivery driver (10)
- ○ Transportation such as Uber or Lyft driver (12)
- ○ Not listed here, please specify: (14) ____

Q19 Does your job require direct contact with patients?

- ○ Yes (1)
- ○ No (2)
- ○ Unsure (3)

End of Block: Demographics

Start of Block: Experiences with COVID-19

Q20 **We would like to learn more about your personal experience with COVID-19:**

In the last month, how often did you talk with other people about COVID-19?

- ○ Every day (1)
- ○ Most days (2)
- ○ Some days (3)
- ○ Never (4)

Q21 How much has worry or stress related to COVID-19 negatively affected your mental health?

- A great deal (1)
- A lot (2)
- A moderate amount (3)
- A little (4)
- None at all (5)

Q22 On a scale of 0 to 10 where 0 corresponds to no fear and 10 corresponds to maximum fear, how afraid are you about getting infected with COVID-19?

	No fear 0	Maximum fear 10
Please select your choice ()		

Q23 On a scale of 0 to 10 where 0 corresponds to no fear and 10 corresponds to maximum fear, how afraid are you about infecting a person close to you (e.g. family member, friend, etc) with COVID-19?

	No fear 0	Maximum fear 10
Please select your choice ()		

Q24 Have you previously been diagnosed with COVID-19 disease?

- Yes, I was diagnosed by a healthcare provider or tested positive (1)
- No, I was not diagnosed but I believe I had COVID-19 (2)
- No, I have never had COVID-19 (3)
- Unsure (4)

Q25 On a scale of 1-10, what was the severity of your illness with COVID-19 disease? 0 corresponds to "I felt no symptoms" and 10 corresponds to "I was hospitalized"

	I felt no symptoms 0	I was hospitalized 10
0 I felt no symptoms – 10 I was hospitalized. ()		

Q26 Did you isolate yourself while you had COVID-19? According to the CDC, isolation means to keep someone who is sick or tested positive for COVID-19 without symptoms away from others, even in their own home.

- Yes (1)
- No (2)

Q27 Have you ever quarantined due to potential exposure to a COVID-19 case or recent travel? According to the CDC, quarantine means to keep someone who was in close contact with someone who has COVID-19 away from others.

- Yes (1)
- No (2)

Q28 Where did you stay while you were isolating or quarantining? (check all that apply)

- [ ] USF dorm room (1)
- [ ] With my parents or other family members (2)
- [ ] In a hotel room (3)
- [ ] In my off-campus apartment (4)
- [ ] Not included here, please specify: (5) ____

---

*

Q29 How many days did you isolate or quarantine for?

____

Q30 Which of the following describes your feelings while isolating or quarantining?

- ○ It was very hard (1)
- ○ It was moderately hard (2)
- ○ It was a little hard (3)
- ○ It was neither hard nor easy (4)
- ○ It was a little easy (5)
- ○ It was moderately easy (6)
- ○ It was very easy (7)

Q31 Did you receive support services from USF during isolation or quarantine?

- Yes (1)
- No (2)

Q32 How did you feel about the USF support services that were provided to you while you were isolating or quarantining?

- It was very helpful (1)
- It was mostly helpful (2)
- It was a little helpful (3)
- It was not at all helpful (4)

Q33 Do you know anyone who has been diagnosed with COVID-19 disease? (check all that apply)

- Yes, a family member (1)
- Yes, a close friend (2)
- Yes, a coworker or colleague (3)
- Yes, someone else (4)
- No (5)

Q34 Do you know anyone who has been hospitalized with COVID-19 disease? (Check all that apply)

- ☐ Yes, a family member (1)
- ☐ Yes, a close friend (2)
- ☐ Yes, a coworker or colleague (3)
- ☐ Yes, someone else (4)
- ☐ No (5)

Q35 Do you know anyone who has died from COVID-19 disease? (Check all that apply)

- ☐ Yes, a family member (1)
- ☐ Yes, a close friend (2)
- ☐ Yes, a coworker or colleague (3)
- ☐ Yes, someone else (4)
- ☐ No (5)

Q36 Since the COVID-19 pandemic began, how many times have you been tested for COVID-19?

If you have been tested many times, please try to estimate how many times you have been tested in total.

____

End of Block: Experiences with COVID-19

Start of Block: Preventive Practices - Behaviours

Q37 **We are going to ask you some questions about your preventive practices related to COVID-19. Please remember that your answers are strictly confidential:**

In the last month, how often did you engage in the following behaviors?

	All the time (1)	Most of the time (2)	Some of the time (3)	A little of the time (4)	Not at all (5)
Staying 6 feet away from people who are not in your household, when indoors (1)	○	○	○	○	○
Staying 6 feet away from people who are not in your household, when in public settings, including outside (2)	○	○	○	○	○
Wearing a mask indoors, with people who are not from your household (3)	○	○	○	○	○
Avoid crowds and poorly ventilated indoor spaces (4)	○	○	○	○	○
Washing your hands or use hand sanitizer frequently (5)	○	○	○	○	○

Q38 How often do you think that <u>your friends</u> practice the following behaviors?

	All the time (1)	Most of the time (2)	Some of the time (3)	A little of the time (4)	Not at all (5)
Staying 6 feet away from people who are not in their household, when indoors (1)	o	o	o	o	o
Staying 6 feet away from people who are not in their household, when in public settings, including outside (2)	o	o	o	o	o
Wearing a mask indoors, with people who are not their your household (3)	o	o	o	o	o
Wearing a mask in public settings, even outside (4)	o	o	o	o	o
Washing their hands frequently (5)	o	o	o	o	o

Q39 Compared to the beginning of the pandemic, would you say that you currently practice physical distancing more or less?

- Definitely more (1)
- Moderately more (2)
- The same (3)
- Moderately less (4)
- Definitely less (5)

Q40 Compared to the beginning of the pandemic, would you say that you currently wear a mask or face covering more or less?

- Definitely more (1)
- Moderately more (2)
- The same (3)
- Moderately less (4)
- Definitely less (5)

Q41 How effective or ineffective do you think the following behaviors are for preventing transmission of COVID-19?

	Very effective (1)	Mostly effective (2)	Somewhat effective (3)	Somewhat ineffective (4)	Mostly ineffective (5)	Very ineffective (6)
Staying 6 feet away from people who are not in your household, when indoors (1)	○	○	○	○	○	○
Staying 6 feet away from people who are not in your household, when in public settings, including outside (2)	○	○	○	○	○	○
Wearing a mask indoors, with people who are not from your household (3)	○	○	○	○	○	○
Wearing a mask in public settings, even outside (4)	○	○	○	○	○	○
Washing your hands frequently (5)	○	○	○	○	○	○

Getting tested for COVID-19 (6)	○	○	○	○	○	○
Getting the COVID-19 vaccine, for myself (7)	○	○	○	○	○	○
Other people getting the COVID-19 vaccine (8)	○	○	○	○	○	○

Q42 When USF returns to in-person learning and living, how confident are you that you can practice the following behaviors if they are recommended by USF?

	Very confident (1)	Somewhat confident (2)	Not too confident (3)	Not at all confident (4)
Wear a mask or face covering during class (1)	○	○	○	○
Wear a mask or face covering when you're with friends inside a campus building (2)	○	○	○	○
Stay 6 feet apart when you are with friends inside a campus building (3)	○	○	○	○
Stay 6 feet away from others during class (6)	○	○	○	○

Q43 How likely do you think you are to get infected with COVID-19 in the next month?

- Very likely (1)
- Somewhat likely (2)
- Not too likely (3)
- Not at all likely (4)

Q44 How likely do you think your friends are to get infected with COVID-19 in the next month?

- Very likely (1)
- Somewhat likely (2)
- Not too likely (3)
- Not at all likely (4)

Q45 How likely do you think your family members are to get infected with COVID-19 in the next month?

- Very likely (1)
- Somewhat likely (2)
- Not too likely (3)
- Not at all likely (4)

End of Block: Preventive Practices - Behaviours

Start of Block: Attitudes about COVID-19 Infection & Preventive Practices

Q46 **The next questions ask about beliefs and attitudes related to COVID-19:**

How strongly do you agree or disagree with the following statements?

	Strongly disagree (1)	Disagree (2)	Neither disagree or agree (3)	Agree (4)	Strongly agree (5)
It's easy to wear a mask while indoors, while with people who are not from your household (1)	○	○	○	○	○
It's easy to wear a mask in public settings, even outside (2)	○	○	○	○	○
It's easy to stay 6 feet away from people who are not in your household, when indoors (3)	○	○	○	○	○
It's easy to stay 6 feet away from people who are not in your household, when in public settings including outside (4)	○	○	○	○	○

Q47 How strongly do you agree or disagree with the following statements?

	Strongly Disagree (1)	Disagree (2)	Neither disagree or agree (3)	Agree (4)	Strongly agree (5)
If I see my friends not wearing masks or keeping their distance, I speak up (1)	○	○	○	○	○
If I see strangers not wearing masks or keeping their distance, I speak up (2)	○	○	○	○	○
I will do everything I can to reduce COVID-19 transmission when I am back on campus by following campus policies (3)	○	○	○	○	○
I really want to get back to "normal." (4)	○	○	○	○	○
I will be shamed by others if I test positive for COVID-19 (5)	○	○	○	○	○

Q48 How strongly do you agree or disagree with the following statements?

	Strongly disagree (1)	Disagree (2)	Neither disagree or agree (3)	Agree (4)	Strongly agree (5)
I am very concerned about someone in my family getting sick from COVID-19 (3)	○	○	○	○	○
The thought of having COVID-19 disease scares me (4)	○	○	○	○	○
I believe that COVID-19 is a serious disease (5)	○	○	○	○	○
There are limits to what I can do to prevent getting the virus (6)	○	○	○	○	○
Wearing a face mask for a long time can be harmful to one's health (7)	○	○	○	○	○
Young adults do not get very sick from COVID-19 disease (8)	○	○	○	○	○
The coronavirus is no worse than the seasonal flu (9)	○	○	○	○	○

The number of deaths from COVID-19 have been exaggerated (10)	○	○	○	○	○
Most people who are diagnosed with COVID-19 will recover (11)	○	○	○	○	○

End of Block: Attitudes about COVID-19 Infection & Preventive Practices

Start of Block: Vaccination

Q49
We would like to learn more about your vaccination behaviors and beliefs. Remember your answers are strictly confidential:

Have you received an influenza (flu) vaccine in the past year?

○ Yes (1)

○ No (2)

○ Unsure (3)

Q50 How frequently do you usually get an influenza (flu) vaccine?

- ◯ Every year (1)
- ◯ Most years (2)
- ◯ Occasionally (3)
- ◯ Once or twice (4)
- ◯ Never (5)

Q51 Have you received a COVID-19 vaccination?

- ◯ Yes (1)
- ◯ No (2)

Q52 Where did you get the COVID-19 vaccination?

- ◯ Doctor’s office, clinic, or health center (1)
- ◯ Workplace or school (2)
- ◯ Health department (3)
- ◯ Mass vaccination site or center (6)
- ◯ Pharmacy or grocery store (4)
- ◯ Other (5) ____

Q53 Did you get the second dose of the COVID-19 vaccine?

◯ Yes (1)

◯ No, but it has been scheduled (2)

◯ No, I do not plan to receive a second dose (3)

Q92 For what reasons do you not plan to receive the second dose of COVID-19 vaccine?

____

Q54 What was your main reason for receiving the COVID-19 vaccine?

◯ My workplace required it (1)

◯ To protect myself (2)

◯ To protect my family and friends (3)

◯ To protect my community (4)

◯ Other (5) ____

Q55 When it becomes available to you, how soon would you like to receive the COVID-19 vaccine?

◯ As soon as possible (1)

◯ I will wait and see how the vaccine is working for other people before I get vaccinated myself (2)

◯ I will get it only if it is required for work, school, athletics, travel or other activities (3)

◯ I definitely will not get the vaccine, even if it is free and determined to be safe by scientists (4)

Q56 For what reasons do you say this?

____

Q57 What is your main reason for planning to receive the COVID-19 vaccine?

- ○ My workplace required it (1)
- ○ To protect myself (2)
- ○ To protect my family and friends (4)
- ○ To protect my community (5)
- ○ Other (6) ____

Q58 What is your main reason for not planning to receive the COVID-19 vaccine?

- ○ I am worried about possible side effects from the vaccine (1)
- ○ I do not think I will be able to access or buy the vaccine (3)
- ○ I do not think I need the vaccine (4)
- ○ Other (5) ____

Q59 Which comes closer to your view: engaging in practices to prevent getting COVID-19 (like mask wearing and physical distancing) is a personal choice OR is part of everyone's responsibility to protect the health of others?

- ○ Personal choice (1)
- ○ Everyone's responsibility (2)
- ○ Neither (3)
- ○ Don't know (4)

Q60 Which comes closer to your view: getting vaccinated against COVID-19 is a personal choice OR getting vaccinated is part of everyone's responsibility to protect the health of others?

- ○ Personal choice (1)
- ○ Everyone's responsibility (2)
- ○ Neither (3)
- ○ Don't know (4)

Q61 How acceptable or unacceptable do you think it would it be for the following organisations to require COVID-19 vaccination (unless a person has a medical reason not to)?

	Definitely acceptable (1)	Moderately acceptable (2)	Moderately unacceptable (3)	Definitely unacceptable (4)
The state or local health department (1)	○	○	○	○
Hospitals and other healthcare sector employers (2)	○	○	○	○
Non-healthcare sector employers (3)	○	○	○	○
University of San Francisco (4)	○	○	○	○

End of Block: Vaccination

Start of Block: Attitudes about COVID-19 Vaccination

Q62 **We would like to learn more about your thoughts on COVID-19 vaccination:**

How strongly do you agree or disagree with the following statements about COVID-19 vaccination?

	Strongly disagree (1)	Disagree (2)	Neither disagree or agree (3)	Agree (4)	Strongly agree (5)
COVID-19 vaccines are safe (1)	○	○	○	○	○
COVID-19 vaccines should be mandatory (2)	○	○	○	○	○
The COVID-19 vaccine will protect me from infection (3)	○	○	○	○	○
I am worried about possible long-term health effects of the COVID vaccine (4)	○	○	○	○	○
The information I receive about COVID-19 vaccines is reliable and trustworthy (5)	○	○	○	○	○
I trust that my government is making decisions in my best interest with respect to the COVID-19 vaccines that were approved (6)	○	○	○	○	○

Reports about side effects from COVID-19 vaccines I have read in the media or on social media have made me worried about getting vaccinated (7)	○	○	○	○	○
I am worried about getting the COVID-19 vaccine because it was approved so quickly (8)	○	○	○	○	○
The development of the COVID-19 vaccine has taken the needs of my community into account (9)	○	○	○	○	○

Q63 How strongly do you agree or disagree with the following statements about COVID-19 vaccination?

	Strongly disagree (1)	Disagree (2)	Neither disagree or agree (3)	Agree (4)	Strongly agree (5)
The COVID-19 vaccine can give you COVID disease (1)	○	○	○	○	○
The COVID-19 vaccine enters your cells and changes your DNA (2)	○	○	○	○	○
The COVID-19 vaccine can affect women's fertility (3)	○	○	○	○	○
If I already had COVID-19, I do not need to get the COVID-19 vaccine (4)	○	○	○	○	○
I don't need to get the COVID-19 vaccine because other people who get it will keep me protected (5)	○	○	○	○	○
I do not need a second dose of COVID-19 vaccine to be fully protected (6)	○	○	○	○	○

I have a good understanding of how the COVID-19 vaccine works (7)	o	o	o	o	o
Getting the COVID-19 vaccine means I can stop wearing my mask and taking coronavirus precautions (8)	o	o	o	o	o

End of Block: Attitudes about COVID-19 Vaccination

Start of Block: Attitudes about Vaccination in General

Q64 **The next few questions ask about your thoughts on vaccines in general.**

How strongly do you agree or disagree with the following statements about vaccines?

	Strongly disagree (1)	Disagree (2)	Neither disagree or agree (3)	Agree (4)	Strongly agree (5)
My religion/philosophy/culture recommends against taking vaccines (1)	○	○	○	○	○
I trust pharmaceutical companies to provide safe and effective vaccines (2)	○	○	○	○	○
Vaccines are important for my health (3)	○	○	○	○	○
Getting vaccinated is important for the health of others in my community (4)	○	○	○	○	○
Generally, I do what my doctor or health care provider recommends about vaccines (5)	○	○	○	○	○
New vaccines carry more risks than older vaccines (6)	○	○	○	○	○
I am concerned about serious adverse effects of vaccines (7)	○	○	○	○	○

End of Block: Attitudes about Vaccination in General

Start of Block: Information Sources

Q65
The next few questions ask about your thoughts on information sources:

Where do you get information about COVID-19? (Check all that apply)

- ☐ Government or quasi-government health agencies, like the Centers for Disease Control and Prevention (CDC), the World Health Organization (WHO), or the local Health Department (1)
- ☐ President Biden (2)
- ☐ Former President Trump (3)
- ☐ My doctor or health care provider (4)
- ☐ News from websites, papers, TV, or magazines (5)
- ☐ Social media (Facebook, Instagram, Twitter, Reddit, etc.) (6)
- ☐ USF COVID-19 Resource Page (7)
- ☐ Professors at USF (8)
- ☐ USF President Father Paul Fitzgerald (9)
- ☐ USF Health Promotion Services (10)
- ☐ USF Student Housing and Residential Education (SHARE) (11)
- ☐ USF Dean of Students' Office (12)
- ☐ President of the USF Student Senate (13)
- ☐ Friends and Family (14)
- ☐ Not listed here, please specify: (15) ____

Q66 How much do you trust the following sources to provide reliable information about COVID-19

	A great deal (1)	A fair amount (2)	Not much (3)	Not at all (4)
Government or quasi-government health agencies, like the Centers for Disease Control and Prevention (CDC), the World Health Organization (WHO), or the local Health Department (6)	○	○	○	○
President Biden (7)	○	○	○	○
Former President Trump (8)	○	○	○	○
My doctor or health care provider (9)	○	○	○	○
News from websites, papers, TV, or magazines (10)	○	○	○	○
Social media (Facebook, Instagram, Twitter, Reddit, etc. (11)	○	○	○	○
USF COVID-19 Resource P (12)	○	○	○	○
Professors at USF (13)	○	○	○	○
USF President Father Paul Fitzgerald (14)	○	○	○	○
USF Health Promotion Services (15)	○	○	○	○

USF Student Housing and Residential Education (SHARE) (16)	○	○	○	○
USF Dean of Students' Office (17)	○	○	○	○
President of the USF Student Senate (18)	○	○	○	○
Other, please specify: (20)	○	○	○	○

End of Block: Information Sources

Start of Block: Additional Demographic and Health Questions

Q67 **Finally, we have a few more questions to ask you to learn a bit more about you:**

What religion or spirituality do you practice?

○ Christian-Catholic (1)

○ Christian-Not Catholic (2)

○ Buddhist (3)

○ Hindu (4)

○ Jewish (5)

○ Muslim (6)

○ Other religion or faith (7)

○ Unaffiliated (8)

Q68 Which of the following best describes you?

- Never been married (1)
- Currently married or living with partner (2)
- Separated, divorced or widowed (3)

Q69 Which one of these comes closest to your feelings about your income?

- Living comfortably on present income (1)
- Getting by on present income (2)
- Finding it difficult on present income (3)
- Finding it very difficult on present income (4)

Q70 Are you currently pregnant?

- Yes (1)
- No (2)
- Unsure (3)

Q71 Do you currently smoke cigarettes?

- Yes, daily (1)
- Yes, sometimes (2)
- No (3)

Q72 Was one or more of your parents born outside the United States?

- ○ Yes (1)
- ○ No (2)
- ○ Unsure (3)

Q73 Do you have health insurance?

- ○ Yes, through school (USF) (1)
- ○ Yes, through employer-sponsored private health insurance (6)
- ○ Yes, through the government (Medicare, CHAMPUS, Tricare, Medicaid/MediCal, Active Military) (2)
- ○ Yes, through an individual insurance plan (including Covered California or other Healthcare Insurance exchange or direct with an insurance company, not affiliated with an employer-based plan) (3)
- ○ Yes, Not listed here (Please specify): (4) ____
- ○ No (5)

Q74 How do you describe your sexual orientation?

- ○ Straight (1)
- ○ Gay (2)
- ○ Lesbian (3)
- ○ Bisexual (4)
- ○ Queer (5)
- ○ My sexual orientation is not included above (6)

Q75 What is your height without shoes on?

- ○ Ft (1) ____
- ○ Inches (2) ____

Q76 How much do you currently weigh?

- ○ Lbs (1) ____

End of Block: Additional Demographic and Health Questions
