# Supplementary material for: High engagement in nonpharmaceutical interventions and their associations with reduced COVID-19 among US college students
Source: BMC Public Health. 2023 May 26;23:971. doi: 10.1186/s12889-023-15916-0 (PMC10214357; doi:10.1186/s12889-023-15916-0)
Supplement: Supplementary file 2 — Additional file 2. [file 12889_2023_15916_MOESM2_ESM.docx]

| **Supplementary Table 1: Adjusted risk ratios of COVID-19 disease by consistent engagement in preventive behaviors among college students (sensitivity analyses)** | | | |
| --- | --- | --- | --- |
|  | **Risk Ratio** |  | **95% CI** |
| **Preventive behavior*** |  |  |  |
| Wear face mask indoors |  |  |  |
| *Inconsistently* | 1.0 |  |  |
| *Consistently* | 0.74 |  | 0.57-0.95 |
| Physical distance indoors |  |  |  |
| *Inconsistently* | 1.0 |  |  |
| *Consistently* | 0.64 |  | 0.49-0.85 |
| Physical distance outdoors/in public settings |  |  |  |
| *Inconsistently* | 1.0 |  |  |
| *Consistently* | 0.74 |  | 0.52-1.03 |
| Avoid crowds/poorly ventilated spaces |  |  |  |
| *Inconsistently* | 1.0 |  |  |
| *Consistently* | 0.81 |  | 0.50-1.30 |
| **Number of preventive behaviors** |  |  |  |
| 0 | 1.0 |  |  |
| 1 | 1.11 |  | 0.47-2.64 |
| 2 | 0.60 |  | 0.26-1.40 |
| 3 | 0.61 |  | 0.27-1.35 |
| 4 | 0.52 |  | 0.24-1.11 |

* Preventive behaviors variables were recoded as “Consistently” including “all the time”

and “most of the time” responses, and Inconsistently” including “some of the time”,

“a little of the time” and “not at all”.
